# Honey authenticity: the opacity of analytical reports—part 2, forensic evaluative reporting as a potential solution

**DOI:** 10.1038/s41538-022-00127-5

**Published:** 2022-02-08

**Authors:** M. J. Walker, S. Cowen, K. Gray, P. Hancock, D. T. Burns

**Affiliations:** 1 Laboratory of the Government Chemist, Queens Road, Teddington, TW11 0LY UK; 2 Institute for Global Food Security, The Queen’s University of Belfast, BT9 5AG Belfast, UK

**Keywords:** Analytical chemistry, Business and industry

## Abstract

The analytical techniques applied to verify honey authenticity are multifaceted and often result in complex data rich certificates of analysis that are open to interpretation and may be opaque to stakeholders without specialist knowledge. In these cases, the drawing of an independent overarching opinion is challenging. Two questions arise: (Q1) Is it acceptable to report interpretation, particularly if it is adverse, without exhibiting the supporting data? (Q2) How may a valid overarching opinion on authenticity be derived from a large, partially conflicting, dataset? To Q1, it is demonstrated that full disclosure of the data used in interpretation is mandatory. To Q2 it is proposed, with worked examples, to adopt ‘evaluative reporting’; a formalised likelihood ratio thought process used in forensic science for evaluation of findings and their strength assessment. In the absence of consensus on *techniques* for honey authenticity adoption of *reporting* conventions will allow objective assessments of reports, with equity to all and provide a better basis to identify and address fraud.

## Introduction

In Part 1 (Honey authenticity: the opacity of analytical reports - part 1, defining the problem) adulteration of honey and methods for its detection were reviewed followed by a detailed examination of 177 individual results from three certificates of analysis (CoA), representative of 13 CoA underpinning a press article^1^ questioning honey authenticity in the UK. The article is a vignette of the difficulties that surround honey and some of the advanced analytical techniques applied to its authentication. The CoA examined alleged some non-compliances, cast suspicion on the authenticity of the honey samples and reported, without detailed findings, the presence of syrup markers and foreign sugars. The findings were based on advanced techniques that result in complex and data rich CoA that are open to interpretation and may be opaque to stakeholders without specialist knowledge. Detailed inspection of the data revealed a nuanced and partially conflicting picture of the authenticity of the samples examined. Thus, two questions arose. (1) Is it acceptable to report interpretation, particularly adverse opinions, without exhibiting the supporting data? (2) How may a valid overarching opinion on authenticity be derived from a large partially conflicting dataset?

In the UK food law is largely criminal law although administrative tools such as improvement notices are used much more commonly than criminal justice proceedings^2^. Economic and societal consequences of food fraud or crime can be severe, as the horse meat scandal of 2013 demonstrated^3–5^, mandating stringent sanctions. Reputational damage from alleged food law contravention may also be severe, for example the reported 15 to 30% decrease in Australian honey sales costing the industry upwards of $10 million following adverse media comment based on NMR results^6^. The criminal burden of proof ‘beyond reasonable doubt’, mandatory for food law enforcement, thus seems appropriate too for less formal reporting. Forensic principles of data interpretation and reporting standards are proposed herein. If adopted, it is believed they will alleviate the potential opacity of reports on honey authenticity, putting in place a more robust basis for interpretation, with equity to all stakeholders and provide a better basis to identify and address fraud should it occur, thus enhancing consumer confidence.

## (1) Is it acceptable to report interpretation, particularly adverse opinion, without exhibiting the supporting data?

Reluctance to disclose data is not new. Many UK Public Analysts (official food control scientists) in the late 19^th^ and early 20^th^ centuries held that detailed analytical results and method details should, as far as possible, be kept out of official certificates tendered in court proceedings. However the view soon emerged from UK court of appeal decisions that it is insufficient for the analyst to give only opinion; there must be sufficient information before the court to allow it to arrive at a conclusion and the defendant(s) should have such information as could enable a rebuttal defence to be advanced if scientifically available^7^. The UK Crown Prosecution Service (CPS) amplifies this in its current legal guidance^8^ on expert evidence which must be on “ …*a sufficiently reliable scientific basis … or … part of a body of knowledge or experience which is sufficiently organised or recognised to be accepted as reliable …”* Moreover, the expert must provide the court with the necessary scientific criteria against which to judge their conclusions and if an inference is given from the findings, an explanation is expected on how safe or unsafe it is and the margin of uncertainty. The UK courts do not reject novel techniques although they must be accredited or sufficiently sound, the factors to be considered are:

“1. *Whether the theory or technique can be or has been tested;*

*2. Whether the theory or technique has been subject to peer review and publication;*

*3. The known or potential rate of error or the existence of standards; and*

*4. Whether the theory or technique used has been generally accepted.”*

The CPS guidance concludes that it is crucial that expert findings are conveyed in a way that is easily understood by the layperson. As a participant in criminal proceedings, the expert has a duty to ensure “that evidence, whether disputed or not, is presented in the clearest and shortest way” (Crim. PR 3.2.2 (e)). Reports should be robust, logical, transparent and balanced^9^.

The above considerations impose a clear obligation to disclose the data on which honey (or any) authenticity findings are based, for example molecular markers or NMR profiles.

## (2) How may a valid overarching opinion on authenticity be derived from a large partially conflicting dataset?

Typically, in reporting results from multiple techniques used to appraise authenticity of honey, much of the data may be unremarkable, some may be apparent evidence of non-compliance with legal requirements, some may raise suspicions, some may indicate likely adulteration, while some are accepted as evidence of adulteration. Data raising suspicions are often accompanied by phrases such as “it is *possible* that …indicates adulteration”, reflecting in part the tentative nature of much of the published literature on honey composition. The recommendations of two recent seminars on honey authenticity^10,11^ are welcomed as are general recommendations on the building and curation of food authenticity databases^12^. However in the shorter-term question 2 is more concerned with the agreed conventions of reporting and of interpretation. Standardised reporting, among other things, has been called for in non-targeted fingerprinting for authentication of food in official controls^13^. A weight of evidence approach has been advocated for honey authenticity evaluation^14^ and is routinely applied, albeit with certain limitations, for example by supplementing analytical findings with mass balance and traceability checks. EFSA has produced guidance on the use of weight of evidence, particularly for chemical risk assessment, and defines a process in which evidence is integrated to determine the relative support for possible answers to a question. The guidance deals with both qualitative and quantitative approaches and describes three basic steps: (1) assembling the evidence into lines of similar type, (2) weighing the evidence, (3) integrating the evidence. EFSA identifies reliability, relevance and consistency as three basic considerations for weighing evidence^15^. However, to our knowledge, detailed guidance on the appraisal of complex multifaceted data on honey authenticity remains to be established. To address this gap, drawing on the application of mainstream forensic science to food authenticity testing^16^ ‘Evaluative Reporting’ is proposed as a means of aligning established forensic best practice and EFSA guidance on weighing and integrating the evidence.

### Evaluative reporting

Evaluative reporting has evolved in mainstream forensic science as a robust framework for interpreting complex data in which a pair of opposing, or competing, propositions are considered. Such propositions exist within a hierarchy of (I) ‘Source’, (II) ‘Activity’ and (III) ‘Offence’^17^. In mainstream forensic science the ‘source’ of evidentiary material, such as glass fragments, fibres or various body fluids, is amenable to (bio)analytical investigation. The probability of the evidence given each proposition can be considered from a comparison between a crime scene sample and a control sample or a population of alternative sources, the analytical characteristics for which might be contained within a reference database which must be relevant to evidential material under investigation. The similarities with analysis of honey are obvious. The breakage of a window, the assault of a person or the adulteration of a honey represent level II ‘activity’ propositions which bring in questions of evidential transfer, persistence, a framework of circumstances and again the relevance of the reference databases. Level III is also an activity level but relates to the actual offence, the legal non-compliance which may be the subject of court proceedings^18^. The paired propositions must be mutually exclusive, exhaustive and avoid ambiguities between the hierarchy levels. Although informal ‘explanations’ (as opposed to propositions)^18^ around whether or not the honey is adulterated are helpful in formulating propositions, in the overall context of honey authenticity objectivity is best served by beginning at level I in the hierarchy. For example, the United States Pharmacopoeia, USP^19^ regards a non-targeted method for detecting food fraud as asking the question: “Is the test sample Typical or Atypical compared to a reference set of Typical samples?” USP adds that a “Typical” outcome does not disprove the presence of adulterants, (e.g. if <LoD (Limit of Detection) or outside the method capability). An “Atypical” outcome suggests that the unknown sample is not consistent with the reference set and could be a truly adulterated sample or an authentic sample with compositional or matrix parameters outside the reference set. A single atypical result does not generally provide a sufficient degree of evidence but should trigger additional analyses. Thus it is proposed that for honey the two competing propositions should be of the form (1) “this honey sample contains compound(s) not found in, or is in some way untypical of, a reference dataset”, and (2) “this honey sample has compositional or matrix parameters outside those represented in the reference set because … (give a reason) or arising by chance”.

The European Network of Forensic Science Institutes, ENFSI 2015, has produced guidance^20^, on evaluative reporting as has the Australia and New Zealand Policing Advisory Agency, ANZPAA 2017^21^. The guidance requires two conditions to be met for the application of evaluative reporting illustrated in Table 1 and translated into food authenticity or food crime investigations. The strength of the evidence is evaluated by considering the ratio of its likelihood given each of the propositions.

**Table 1:** Evaluative reporting conditions.

Mainstream forensic science	Food crime
1. The forensic practitioner has been asked by a mandating authority or party to examine and/or compare material (typically recovered trace material with reference material from known potential sources)	As in mainstream forensic science, when the circumstances are equivalent, although this may be rare^33^. More often samples are compared with a supposedly ‘genuine’ (‘typical’) article, or more usually a dataset representing certain characteristics of a ‘typical sample’.
2. The forensic practitioner seeks to evaluate findings with respect to particular competing propositions set by the specific case circumstances or as indicated by the mandating authority.	The competing propositions may be (1) the sample is an ‘atypical’ product or (2) the sample is a ‘typical’ of an authentic sample with compositional or matrix parameters outside that represented in the reference set.

### Likelihood ratio

A likelihood ratio (LR) is a ratio of probabilities expressing the relative likelihood of observing the evidence *E* under two competing propositions:

1 $$LR = \frac{{PE|Hp}}{{PE|Hd}}$$

In Eq. (1) *H*_*p*_ and *H*_*d*_ represent the two propositions (‘prosecution’ and ‘defence’) and the probability *P*, of obtaining the evidence *E* is conditional on each proposition. Thus a high LR corresponds to a situation in which it is much more likely that the observed result (the ‘evidence’ *E*) would occur if *H*_*p*_ were true rather than *H*_*d*_. This represents strong evidence in support of *H*_*p*_, though it is important to note that a high LR alone says nothing about the probability that *H*_*p*_ is actually true, (a decision for the court to take on *all* the evidence before it). In order to make a statement about the probability of *H*_*p*_ given *E* (the ‘transposed conditional’ see below), additional information is needed in the form of a prior probability for *H*_*p*_. The choice of prior probability is highly influential on the outcome, and for this reason is not considered suitable as it introduces a subjective aspect into what should be an objective statement. Evidence is instead presented in the form of the ‘weight of evidence’ calculation as represented by Eq. (1) without considering prior probabilities. The relative strength of the evidence is articulated from a generally agreed verbal scale and equivalent likelihood ratio bands, Table 2. The choice of the reported verbal equivalent is based on the likelihood ratio and not the reverse.

**Table 2:** Forensic verbal scale.

Supported proposition	Verbal scale		LR (likelihood ratio)
	ENFSI	Literature, e.g. Marquis et al.^34^	
First proposition is supported against the alternative proposition		Null	1
	Slight support /Limited support	Weak or limited support	1 < LR ≤ 10
	Moderate support	Moderate support	10 < LR ≤ 100
	Moderately strong support	Strong support	100 < LR ≤ 1000
	Strong support	Very strong support	1000 < LR ≤ 10,000
	Very strong support	Extremely strong support	LR > 10,000

Particular care must be taken to avoid ‘transposing the conditional’ also known as ‘the prosecutor’s fallacy’ which confuses the probability of particular findings given a proposition with the probability of that proposition given these findings. Evett in 1995^22^ gave a trivial example: the probability, *P*, that an animal has four legs if it is a cow is *P* = 1. Stating with *P* =1 that an animal is a cow if it has four legs is clearly erroneous. More to the point, the probability, *P*, that a substance is an aqueous solution equimolar in glucose and fructose if it is honey is *P* = 1 but it is wrong to state *P* = 1 that a substance that is an aqueous solution equimolar in glucose and fructose is honey.

The use of probability and LR has been explored in relation to the authenticity of wine (Martyna et al. 2014)^23^ and olive oil (Własiuk et al. 2015)^24^. Vander Zanden and Chesson (2017)^25^ discussed common statistical approaches from the ecological literature to the forensic interpretation of IRMS isotopic data for food authenticity and geographic origin. They considered probabilistic methods, particularly LR, one of the most promising and powerful approaches for IRMS isotope data interpretation for food authenticity.

Application of probabilistic and LR methods to the interpretation of honey authenticity or origin requires dedicated research^26^, not least because some of the datasets are currently part of the debate. In the current absence of such research, the forensic verbal scale is a means to assess findings in an objective manner because, when there are insufficient data, the LR approach provides a framework for structured and logical reasoning based on experience. This holds true so long as the grounds for the opinion can be explained and accompanied by the appropriate degree of understanding of the particular parameter and analytical method.

Whether from databases or experience, reports on findings of honey analysis should be based upon sound science and capable of sustaining scrutiny and cross examination. Transparency requires from the outset a demonstrable process traceable in the sample documentation, case file and report. The latter should be written so that it is suitable for a wide audience of readers and may include supplements explaining the technology and technical findings.

Although an evaluative report necessarily contains technical elements, CoA which do not contain interpretation must be clearly badged as intelligence, investigative or technical reports, not intended of themselves to be conclusive as to authenticity.

## Examples of evaluative reporting

### Diastase

The provisions of EU Directive 2001/110/EC on honey require, with certain derogations inapplicable here, a product described as honey to exhibit a diastase activity (number, DN) of not less than 8 DN. There may be reasons such as time related or thermal decomposition for a lower diastase activity in a particular sample, and at level II or III in the hierarchy of propositions some consideration of authenticity will arise perhaps contingent on other circumstances. Thus at level I the propositions might be (1) *H*_*p*,_ the honey contains diastase activity untypical of official levels and (2) *H*_*d*,_ the honey contains diastase activity consistent with its temporal and/or thermal history. It is tantamount to axiomatic that DN ≤ 8 would be observed for a randomly taken sample of honey non-compliant with the Directive provisions. Hence:

2 $$P\left[ {DN \le 8|H_p} \right] = 1$$

For *H*_*d*_ it is not possible to assign a probability or probability density to a *specific* value of DN without detailed knowledge of the background distribution of DN values which would be associated with samples drawn from the population of genuine samples. However Bogdanov et al., 1999^27^ reported more than 92% of bee keeper honey samples (*n* = ~20,000) and more than 88% of retail honey samples (*n* = ~1000) had a DN greater than 8, supporting the EU Directive lower limit of 8 DN. Thus if a random genuine sample is taken from bee keeper honey on (100-92) = 8% of occasions it might have DN < 8, a probability of *P* = 0.08. On (100-88) = 12% of occasions when a random genuine sample of honey is taken in retail premises it might have a DN < 8, a probability of *P* = 0.12. Thus *H*_*d*_ can be stated:

3 $$P\left[ {DN\, < \,8|H_d} \right] = 0.08$$

4 $$P\left[ {DN\, < \,8|H_d} \right] = 0.12$$

Inserting expressions (2) and (3) or (4) into Eq. (1) yields likelihood ratios, LR, of 12.5 or 8.3 respectively. Referring to Table 2 the results reported for the honeys discussed in Part 1 of this study (Table 1, Honey authenticity: the opacity of analytical reports - part 1, defining the problem) of 4.4, 6.0 and 6.0 DN provide slight, limited or weak support for the proposition that they are untypical of the dataset that gave rise to the limit. In the face of this it is very unlikely, unless other evidence can be adduced, that proceeding to level II or Level III propositions on adulteration or non-compliance can be pursued. Strictly speaking LR =12.5 is in the ‘moderate’ band but the demarcation should be treated conservatively and as shown the LR outcome tolerates some variance in the underpinning data.

In the absence of a LR, a narrative opinion could be given such as:

Certain unifloral honeys have a naturally low diastase activity, the honey Directive stipulates diastase activity is “ … determined after processing and blending” which can be interpreted to mean DN are not valid during the whole shelf life of honey and concern has been expressed that the diastase limit is not met in retail honey during the shelf life of the product, especially during storage at higher temperature^28^.

It can be seen therefore that the LR approach in evaluative reporting enables the strength of evidence for honey authenticity to be considered within a well-recognised, formalised forensic thought-process that is more concise than a narrative opinion. The narrative opinion could, of course, supplement the LR and verbal scale albeit it is considerably less easy to reach a convincing conclusion from a series of results and narrative opinions on the same sample.

### Caramel E150c/d

Table 3 in part 1 (Honey authenticity: the opacity of analytical reports - part 1 defining the problem), reported caramel as detected without giving a LoQ (Limit of Quantitation). In the literature caramel > LoQ (given as 5 mg/kg) is considered non-compliant, added to mimic dark forest honey^29^ and caramel may also arise on heating starch syrups. Therefore, the two propositions might be: *H*_*p*_: the sample is atypical owing to the presence of caramel, thus

5 $$P\left[ {C \ge 5mg/kg|H_p} \right] = 1$$

and, for the purposes of illustration *H*_*d*_: the concentration of caramel (*C*) found in the sample arose inadvertently or by chance. The reporting laboratory’s interpretation included the phrase “it is *possible* (our emphasis) the product has been adulterated” and it may be possible to conceive other explanations for the presence of detected caramel, e.g. (just conceivably) from smoke used to control the bees when managing the hive. In this example a study to illuminate the potential probability of the occurrence of caramel >5 mg/kg appears lacking. In-house experience may give some background information and as the finding is badged as tentative let, say

6 $$P\left[ {C \ge 5mg/kg|H_d} \right] = 0.1$$

i.e. 10% of genuine honey samples may contain caramel and thus LR =1/0.1 = 10. To provide moderate support (LR > 100) for the evidence for *H*_*p*_, it must be supposed (or known) that only 1% of honey might naturally contain caramel and if strong to very strong support (LR > 1000) is required, then it must be supposed (or known) that 0.1% (1 in 1000) or fewer random samples of honey naturally contain caramel. However, in the absence of an appropriate database, the strength of the caramel evidence for *H*_*p*_ clearly remains weak. Similar considerations apply to the results for psicose and honey foreign alpha-amylase reported in the CoA discussed in ‘Honey authenticity: the opacity of analytical reports - part 1 defining the problem’.

### HRMS and NMR

If the nature of alleged HRMS markers and their prevalence in syrups or honey are not disclosed it is difficult to calculate a LR for the competing propositions. However, if it is supposed that a reasonable number of syrups used to adulterate honey have been analysed and the marker(s) occurred in each one, this may enable us to state:

7 $$P\left[ {{{{\mathrm{marker}}}}\, > \,LoQ|H_p} \right] = 1$$

Consider 1000 random ‘genuine’ honey samples analysed with no evidence of marker(s) present, this can be stated

8 $$P\left[ {{{{\mathrm{marker}}}}\, > \,LoQ|H_d} \right] = 0.0009$$

(i.e., less than 1 in 1000 random samples of honey were found to contain the marker). Hence for a sample in which the marker(s) were found LR = 1/0.0009 i.e. ~1100, which would represent strong or very strong support for the proposition that that sample was untypical (and hence level II propositions on adulteration could be pursued), but if 1% (10 in 1000) of random ‘genuine’ honey samples contain marker(s) the LR reduces to below 100 with only moderate support for adulteration.

This gives an indication of the size of the necessary dataset of genuine honeys that would need to be analysed and the data published to render the HRMS marker approach forensically robust. The dataset must also be representative of the honey global supply chain in the particular market where the sample is taken to assess its authenticity.

Similar considerations apply to NMR results. Although there are private databases supporting honey NMR analyses that contain data on >20,000 honey samples^6^ from which, given access to their metadata, it might be possible to construct probability data for the opposing positions as to ‘typical’ or ‘atypical’ honey.

### Combining LR

It is possible to consider a series of findings: a, b, c …*n*, representing similar pairs of opposite propositions (this sample A, B, C, … *N*) is untypical or typical) with LR_a_, LR_b_, LR_c_ … LR_*n*_. How appropriate is it to multiply the LR as a way of aggregating the series of findings into something more meaningful? Clearly if each LR is 0 or 1, nothing changes, a series of inconclusive data does not accumulate into anything meaningful. However consider LR_a_ = LR_b_ = LR_c_ = 5 or, say, 9, their multiplication results in LR = 125 or LR = 729 moderately strong or strong support for the proposition. Combining likelihood ratios is however potentially difficult, as a well-known miscarriage of justice poignantly reveals^30^. It depends essentially on the independence or orthogonality of the findings. If it can be accepted that the findings are fully independent, that is, that the result obtained leading to the first finding has no influence on the results of the other analyses, multiplication of the LRs is appropriate. Lack of independence is often less clear and must be considered carefully and will, with regard to HRMS and NMR, require further research and stakeholder dialogue.

One conservative approach to the non-independence problem is to assume (when circumstances permit) that there are grounds to suppose that result ‘a’ is strongly correlated with result ‘b’. Then, rather than including LR_a_ x LR_b_, only LR_a_ or LR_b_ should be included in the combination. It would be justifiable to choose the larger of the two.

## Conclusions

For the three CoA examined in ‘Honey authenticity: the opacity of analytical reports - part 1, defining the problem’, the interpretation (given current disclosure) can be classified as providing only weak or limited support to the propositions that the honey samples are untypical. The implication is that further propositions on the authenticity of the honey samples do not merit consideration unless further evidence arises.

Appraising the authenticity of honey remains difficult despite decades of research. Of the highly sophisticated approaches more recently deployed, only Elemental Analysis-Liquid Chromatography-Isotope Ratio Mass Spectrometry (EA-LC-IRMS) has gained widespread acceptance owing to use by multiple scientific communities and transparent international metrological assessment, validation and proficiency testing^31,32^. HRMS for syrup markers and NMR have been criticised because of doubts about the databases that underpin the interpretation of their findings in relation to honey^11^. Recommendations herein build on guidance on building and curating food authenticity databases^12^ with an indication of the numbers of samples needed to characterise the evidence as strong.

Transparent reporting in an independent framework that gives a clear indication of the strength of the evidence is the best way to put in place measures to identify and eliminate frauds when they occur. The following recommendations are made for a reporting convention transparently to assess the honey supply chain and thus ensure its integrity.

Dealing with multicomponent results of honey analysis by way of a CoA must:

Begin before the report is drawn up (and preferably before the work is undertaken) with a consideration of two explanations for any findings represented by two alternative propositions of the form

- “this honey sample contains compound(s) not found in, or is [in some specified way] untypical of, a reference dataset”, and
- “this honey sample has compositional or matrix parameters outside those represented in the reference set because … [give a reason] or arising by chance”

1. Reporting of honey data must include sufficient information to enable an impartial observer to interpret each datum, including (rather than a simple ‘positive’), quantitative data, the LoD and/or LoQ, the measurement uncertainty and an indication of the strength of the findings based on an estimate of the likelihood ratio given two propositions of the sort illustrated above.
2. If the strength of the findings is not given the report must be clearly marked as not capable of supporting a definitive judgement about the samples, and is being provided for ‘intelligence’, ‘investigative’ or ‘technical’ purposes so that further work can be undertaken.

In general, there must be disclosure of the studies that generated the probability of the evidence and, if not on the CoA itself, then in the peer reviewed literature, of the datasets that underpin interpretation. If protection of intellectual property or fears that release of information may enable fraudsters to subvert the tests, then confidential release to an appropriate independent body scientifically capable of appraising the data (for example, the UK Government Chemist) might be sufficient.

Further work is needed to assess the databases underpinning physicochemical parameters such as the prevalence of caramel, psicose, certain enzymes, and HRMS markers and NMR. Research is also needed on the independence of the techniques to assess the validity of LR multiplicative combination. Training will be required on the application of probability estimates and likelihood ratios to honey authenticity to facilitate the implementation of evaluative reporting.

## Data Availability

All data generated or analysed during this study are included in this published article (and/or its supplementary information files).
